# The validity and reliability of the interviewer-administered EQ-5D-Y-3L version in young children

**DOI:** 10.1186/s12955-023-02100-6

**Published:** 2023-02-22

**Authors:** Razia Amien, Desiree Scott, Janine Verstraete

**Affiliations:** 1 Division of Physiotherapy, Faculty of Health and Rehabilitation Sciences, University of Cape Town, Cape Town, South Africa; 2 Division of Pulmonology, Department of Paediatrics and Child Health, University of Cape Town, Cape Town, South Africa

**Keywords:** Children, Youth, Interviewer-administered, Self-complete, Health-related Quality of Life, EQ-5D-Y

## Abstract

**Objectives:**

The aim of this study was to determine the validity and reliability of the EQ-5D-Y-3L interviewer-administered (IA) version in South African children aged 5–7-years compared to 8–10-years.

**Methods:**

Children aged 5–10-years (n = 388) were recruited from healthcare facilities, schools for learners with special educational needs and mainstream schools across four known condition groups: chronic respiratory illnesses, functional disabilities, orthopaedic conditions and the general population. All children completed the EQ-5D-Y-3L IA, Moods and Feelings Questionnaire (MFQ), Faces Pain Scale-Revised (FPS-R) and a functional independence measure (WeeFIM) was completed by the researcher. Cognitive debriefing was done after the EQ-5D-Y-3L IA to determine comprehensibility. Test–retest of the EQ-5D-Y-3L IA was done 48 h later and assessed using Cohen’s kappa (k).

**Results:**

Results from children aged 5–7-years (n = 177) and 8–10-years (n = 211) were included. There were significantly higher reports of problems in the *Looking After Myself* dimension in the 5–7-year-olds (55%) compared to the 8–10-year-olds (28%) (*x*^2^ = *31.021*; *p* = *0.000*). The younger children took significantly longer to complete the measure (Mann-Whitney U = 8389.5, *p* < 0.001). Known-group validity was found at dimension level with children receiving orthopaedic management reporting more problems on physical dimensions across both age-groups. Convergent validity between *Looking After Myself* and WeeFIM items of self-care showed moderate to high correlations for both age-groups with a significantly higher correlation in the 8–10-year-olds for dressing upper (z = 2.24; *p* = 0.013) and lower body (z = 2.78; *p* = 0.003) and self-care total (z = 2.01; *p* = 0.022). There were fair to moderate levels of test-retest reliability across age-groups.

**Conclusion:**

The EQ-5D-Y-3L IA showed acceptable convergent validity and test–retest reliability for measuring health in children aged 5–7-years. There was more report of problems with the dimension of *Looking After Myself* in the 5–7-year group due to younger children requiring help with dressing, including buttons and shoelaces due to their developmental age, rather than their physical capabilities. Therefore, it may be useful to include examples of age-appropriate dressing tasks. There was further some reported difficulty with thinking about the dimensions in the younger age-group, most notably for *Usual Activities* which includes a large number of examples. By decreasing the number of examples it may reduce the burden of recall for the younger age-group.

## Background

Health-related Quality of Life (HRQoL) is a multidimensional subjective measure of physical and psychosocial factors in the context of an individual’s daily life [1]. The interest in HRQoL in the paediatric population has grown over the last three decades with an increase in the development of generic preference-based patient-reported outcome measures (PROMs) [2–5]. The information generated from the self-completed PROMs can be used to guide healthcare professionals in tailoring and monitoring treatment interventions [6, 7], to inform population health and clinical research studies and aid decision-making and health technology assessment [6]. The first preference-based value sets of the EQ-5D-Y-3L have been published [8–10] following the international protocol [11]. This will allow for increased use of the EQ-5D-Y-3L to support decision-making and for health technology assessment [6].

The EQ-5D-Y-3L is currently recommended for self-complete from the age of 8-years [12]. The dimensions on the proxy version performed well in children aged 5-years, indicating that it is developmentally appropriate from this age, whereas younger children’s health should be measured on a different instrument [13–15]. Despite the increase in PROM development for the paediatric population, the modes of administration remain limited, especially in younger children who understand the concept of health [16] but may not have the necessary literacy skills to self-complete and therefore have to rely on proxy-report for which we know there is often a mismatch between children and parents [13, 17–19], most noticeably in the psychosocial dimension of feeling *Worried, Sad or Unhappy* [20].

Studies have however suggested that children as young as 5-years with varying health conditions can reliably report their HRQoL with interviewer assistance [21, 22]. Canaway and Frew [23] found the interviewer-administered CHU-9D and EQ-5D-Y-3L to be feasible in children aged 6–7-years but recommended further research to determine the validity and reliability. The EuroQol group recently developed a standardised script to allow for interviewer administration of the EQ-5D-Y-3L. An interviewer-administered version of the EQ-5D-Y-3L has since been developed with a standardised script and instructions for the interviewer. Considering the young age of the sample, respondent burden was a concern and the study aimed to investigate only one of these instruments. The EQ-5D-Y self-complete measure has previously been validated in South Africa in children aged 8–15-years and was thus considered appropriate for further testing in the younger age-group.

The need for more interviewer-administered versions for younger children have become increasingly important to allow children the opportunity to self-report their HRQoL instead of defaulting to proxy-report. The aim of this study was thus to determine the validity and reliability of the EQ-5D-Y-3L interviewer-administered (IA) version in children aged 5–7-years, compared to children aged 8–10-years.

## Methods

### Study design and participants

A cross-sectional, descriptive observational design with repeated measures for test–retest reliability was conducted in children aged 5–10-years in the Western Cape, South Africa. Three research settings, each with children in different health states, but from similar socio-economic backgrounds (low to middle income) were used in Cape Town, South Africa. Children attending two mainstream schools, with generally healthy learners, were used to recruit a general population sample. Children with a functional disability were recruited from three schools for learners with special educational needs. These schools have specialised education services for learners with normal intellect diagnosed with a functional disability (e.g. cerebral palsy, spina bifida or muscle disease). Children with a chronic respiratory illness were recruited at routine outpatient visits at a tertiary paediatric hospital. Children requiring acute medical treatment post fracture or orthopaedic surgery were recruited from the outpatient fracture clinic or the inpatient wards of two paediatric hospitals. All English-speaking children aged 5–10-years, at each facility were eligible for the study. Only children with signed consent and assent forms were included in the study. Those who had a medically diagnosed hearing impairment or cognitive impairment diagnosed by a doctor were excluded as they may have had difficulty with participating in the interview or understanding the measures. Medically unstable children were excluded as the research may have been too distressing.

## Instruments

### EQ-5D-Y-3L

The South African English EQ-5D-Y-3L IA version was used in this study. The EQ-5D-Y-3L consists of five dimensions namely *Mobility* (walking about), *Looking After Myself* (washing and dressing), doing *Usual Activities* (going to school, hobbies, sports, playing, doing things with family or friends), having *Pain or Discomfort* and feeling *Worried, Sad or Unhappy*. Each dimension has three levels of report categorized as level 1 indicating ‘no problems’, level 2 indicating ‘some problems’ or level 3 indicating ‘a lot of problems’ [24]. The EQ-5D-Y-3L includes a Visual Analogue Scale (VAS) which is a vertical, graduated number scale from worst-imagined health state (0) to best-imagined health state (100) on which the participant rates their overall health status also on the day of testing [25, 26].

There are very few generic HRQoL measures that have been validated for use in the South African population. As the study objectives were to compare performance between age-groups comparative data was favourable. As such instruments for comparison to the EQ-5D-Y were drawn from the study by Scott et al. [27] and detailed below.

### Faces Pain Scale-Revised (FPS-R)

The Faces Pain Scale-Revised (FPS-R) is a self-report measure intended to determine the intensity of pain felt by children on the day of testing. It includes a series of six facial expressions depicting an increase in pain intensity from left to right with scores ranging from 0 to 10, increasing by increments of 2. It can be used to self-rate pain intensity in children aged 4-years or older [28].

### Moods and Feelings Questionnaire (MFQ)

The Moods and Feelings Questionnaire (MFQ) consists of 13 questions about the child’s psychological wellbeing in the two weeks before testing. Participants were asked to answer questions on a scale of ‘not true’, ‘sometimes’ or ‘true’. The measure has been found valid and reliable in an international study in children from age 5-years [29].

### WeeFIM

The WeeFIM is an observational instrument used to assess functional independence in children [30, 31]. Functional performance was measured across three dimensions, namely self-care, mobility and cognition. The 18 items are each rated on an ordinal scale from 1 to 7. The scale gives scores for sub-scales (mobility, cognition and self-care) or a total score for functional performance, the higher score, the more independent the child is considered to be in that dimension.

### Cognitive debriefing

A cognitive debriefing guide was developed to determine the comprehensibility of the instrument. The structured script allowed for probing the child to determine the reason behind their answer for each of the dimension scores, e.g. ‘why did you say you have a lot of problems with *Mobility*?’ The cognitive debriefing further aimed to identify any potentially difficult or confusing words used in the EQ-5D-Y-3L [32].

### Procedure

Approvals were granted by the Faculty of Health Sciences, Human Research Ethics Committee, University of Cape Town (HREC 369_2020), ministerial permission for non-therapeutic research with minors, Western Cape Education Department, the respective school principals and the management from healthcare facilities. The study was carried out following the declaration of Helsinki involving human participants [33] and the recommended COVID precautions and restrictions set out by the local government.

Information leaflets detailing the study were sent home with eligible learners at the mainstream schools and schools for learners with special educational needs. Children attending outpatient clinics were recruited on the day of their routine appointments and those admitted to the inpatient setting were recruited from the ward. Those parents who were willing to allow their child to participate completed signed informed consent and demographic information. Children were interviewed in a private room after providing assent. They completed the EQ-5D-Y-3L IA (timed), FPS-R and MFQ in random order. The cognitive debriefing of the EQ-5D-Y-3L IA version followed the completion of the instrument, and the researcher scored the WeeFIM. Children with a functional disability and those from the general population completed a second EQ-5D-Y-3L IA 48 h later, by the same interviewer, to determine test–retest reliability. The time interval of 48 h was proved to be suitable as it is a long enough period for children with a stable health condition not to remember their initial score [34] and short enough to ensure no health related changes occurred in this heterogenous sample [13]. There are no clear guidelines on the most appropriate time period between test–retest for reliability and Marx et al. [34] found no difference between 2 days and 2 weeks.

### Data Management and Analysis

As the EQ-5D-Y-3L self-complete version has been successfully tested for validity, reliability and responsiveness in South African children aged 8–10-years [35], this study compared the performance of the EQ-5D-Y-3L IA in children 5–7-years with those aged 8–10-years [19, 27, 36, 37]. The sample size was considered for each psychometric property in accordance with the COSMIN guidelines where n > 100 per group is considered very good for convergent validity and reliability [38].

### General performance and feasibility

The EQ-5D-Y-3L responses and descriptive data were summarised in terms of the frequency of responses. The ceiling effect of the EQ-5D-Y-3L was defined as the proportion of children scoring no problems in all five dimensions (11,111) or for each dimension. The number of unique health states was computed across age-groups and condition groups. Differences in reporting were determined by chi-square statistic (*x*^2^). The median time taken to complete the EQ-5D-Y-3L IA between the two age-groups was compared with the Mann–Whitney U-test.

### Known-group validity

Known-group validity of the EQ-5D-Y IA was examined by comparing the dimension responses by known health condition i.e. orthopaedic conditions, chronic respiratory illnesses, functional disability and from the general population. Following the methodology used by Ravens-Sieberer [19], the dimension responses were collapsed into ‘no problems’ (level 1) and problems (level 2 and 3 combined) and compared using the Chi-squared test (*x*^2^). The Kruskal–Wallis H test was computed for comparison of VAS scores between groups. It was expected that children with an orthopaedic condition and those with a functional disability would report more problems in the *Mobility* dimension compared to other groups [25, 27, 39]. It was also anticipated that children with an orthopaedic condition (being more acutely ill), would report more problems with *Usual Activities* and *Pain or Discomfort* [27, 40]. Lastly, it was expected that all children with a health condition (orthopaedic, chronic respiratory illness and functional disability) would report greater feelings of *Worried, Sad or Unhappy* than children from the general population [27, 40].

### Convergent validity

The convergent validity of the dimension scores of the EQ-5D-Y-3L IA was compared to the corresponding scores from the WeeFIM, FPS-R and MFQ using Spearman correlations (r_s_). Correlation coefficients were compared between age-groups using the Fisher r-to-z transformation (http://vassarstats.net). Spearman correlation coefficients were interpreted as: 0.1–0.29 low association, 0.3–0.49 moderate association and ≥ 0.5 high association [41].

### Test–retest reliability

Test–retest reliability was assessed using weighted Cohen’s kappa statistic (k) for dimension scores and the Intraclass Correlation Coefficient (ICC) for VAS scores across the two age-groups. As the ICC gives a combined result for intra-observer and inter-observer variability, it is not always easy to interpret and thus Fleiss Kappa (k) and Kendall’s coefficient of concordance (W) have been computed for comparison of VAS scores for interpretation [42].

Cohen’s Kappa and Fleiss Kappa values were interpreted according to Landis and Koch’s guidelines: < 0.2 poor agreement, 0.21–0.40 fair agreement, 0.41–0.60 moderate agreement, 0.61–0.80 substantial agreement [43]. An ICC of > 0.7 was considered reliable [44]. Kendall’s coefficient (W) was interpreted as 0 no agreement, 0.10 weak agreement, 0.30 moderate agreement, 0.60 strong agreement, 1 perfect agreement [45].

### Cognitive debriefing

Qualitative data collected from participants regarding reasons for level reported understanding and inconsistencies were tabulated.

All data analyses were conducted using SPSS Windows 27.0 (IBM SPSS Inc., Chicago, IL, USA) and Statistica Windows Version 13.0 (TIBCO Software Inc., Palo Alto, CA, USA).

## Results

**Fig. 1:**
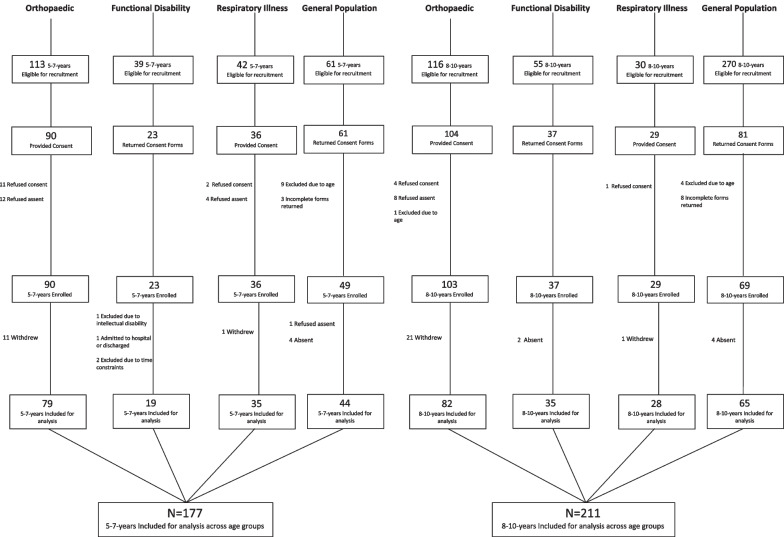
Recruitment of children aged 5–7-years and 8–10-years

The recruitment of children aged 5–7-years and those aged 8–10-years is shown in Fig. 1. There was a high proportion of non-responders in the 5–7-year-olds (n = 78, 44%) and the 8–10-year-olds (n = 260, 55%). The reason for not wanting to participate was not recorded. The number of participants who withdrew was higher in the 8–10-year-old group (n = 21, 20%) compared to the 5–7-year-old group (n = 11, 12%). All participants who withdrew did so due to personal reasons, transport issues, multiple medical appointments and/or time constraints and not for reasons related to the study.

A total of 388 children were recruited across 5–7-years (n = 177, 46%) and 8–10-years (n = 211, 54%). There was no difference between sex (*x*^2^ = 2.34, *p* = 0.126) or health condition (*x*^2^ = 7.21, *p* = 0.065) across age-groups. Health conditions across age-groups are detailed in Table 1.

**Table 1:** Descriptive statistics of participants across age-groups (5–7-years and 8–10-years)

	5–7-years		8–10-years	
	n	%	n	%
Sex	(n = 177)		(n = 211)	
Female	96	54%	98	46%
Male	81	46%	113	54%

Orthopaedic	(n = 79)		(n = 82)	
Upper Limb Fracture	34	43%	31	38%
Lower Limb Fracture	24	30%	21	26%
Surgical correction of acquired or congenital orthopaedic condition^*#*^	17	22%	21	26%
Other*	4	5%	9	11%

Functional disabilities	(n = 19)		(n = 36)	
Cerebral Palsy	8	42%	6	17%
Spina Bifida	3	16%	5	14%
Development Co-ordination Disorderˆ	8	42%	23	64%
Developmental Delay	0	0%	2	6%

Respiratory	(n = 35)		(n = 28)	
Atopy	6	17%	12	43%
Cystic fibrosis	13	37%	5	18%
Bronchiectasis	4	11%	3	11%
Acute respiratory Illness	3	9%	0	0%
Other^*¥*^	9	26%	8	29%

General population	(n = 44)		(n = 65)	
None	40	91%	54	83%
Atopy	3	7%	9	14%
Other^*§*^	1	2%	2	3%

^#^Includes Blount’s disease, osteogenesis imperfecta, developmental dysplasia of the hip, leg length discrepancy and spinal deformity *includes osteitis, septic arthritis and a traumatic amputation. ˆincludes learning disability and Human Immunodeficiency Virus. ^¥^includes damage to the lungs post-acute viral infection, congenital abnormalities of the respiratory system and idiopathic pulmonary haemorrhage. ^§^includes Osteogenesis imperfecta and a congenital cardiac defect

### General instrument performance and feasibility

**Fig. 2:**
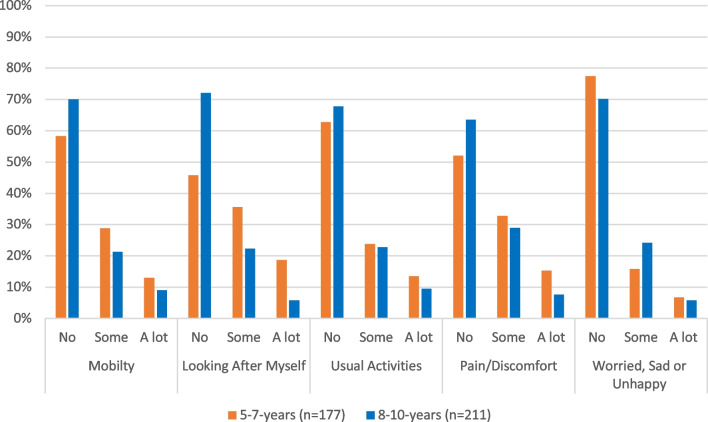
Comparison of the EQ-5D-Y-3L dimensions across age-groups

There were significantly higher reports of some problems and a lot of problems in the dimension of *Looking After Myself* in the 5–7-year-olds (*x*^2^ = 31.021, *p* < 0.001) and *Pain or Discomfort* in the 8–10-year-olds (*x*^2^ = 7.775, *p* = 0.020) (Fig. 2). There were no significant differences in *Mobility* (*x*^2^ = 5.563, *p* = 0.062), *Usual Activities* (*x*^2^ = 1.830, *p* = 0.401), and *Worried, Sad or Unhappy* (*x*^2^ = 4.173, *p* = 0.124), across age-groups.

There was no significant difference in the ceiling effect between the 5–7-year-olds (n = 51, 29%) and the 8–10-year-olds (n = 64, 30%) (*x*^2^ = 0.08, *p* = 0,778). The total reporting of unique health states was significantly higher in the 8–10-year group (n = 111, 53%) than the 5–7-year group (n = 66, 37%) (*x*^2^ = 8.5, *p* = 0.004).

Although the 5–7-year-olds took significantly longer to complete the measure (median = 134 s, IQR = 118, 157) compared to the 8–10-year-olds (median = 110 s, IQR = 98, 125) (Mann–Whitney U = 8389.5, *p* =  < 0.001), both instruments were completed within two and a half minutes.

### Known-group validity

The known-group validity of the EQ-5D-Y-3L IA scores by age and health condition (orthopaedic conditions, chronic respiratory illnesses, functional disabilities and the general population) is shown in Table 2. There was a significant difference between health conditions for the dimensions of *Looking After Myself* and *Usual Activities* in the 5–7-year group*.* All dimensions except *Worried, Sad or Unhappy* were significantly different in the 8–10-year group.

**Table 2:** Known-group validity of EQ-5D-Y-3L by age and health condition

		5–7-years						8–10-years					
		FD	GP	Ortho	Resp	*x*^2^	*p*-value	FD	GP	Ortho	Resp	*x*^2^	*p*-value
		n = 19	n = 44	n = 79	n = 35			n = 36	n = 65	n = 82	n = 28		
Mobility	No problems	53%	66%	49%	71%	6.368	0.095	61%	89%	59%	68%	17.871	** < 0.001**
	Problems	47%	34%	51%	29%			39%	11%	41%	32%		
Looking After Myself	No problems	53%	66%	34%	43%	11.947	**0.008**	67%	89%	54%	93%	29.83	** < 0.001**
	Problems	47%	34%	66%	57%			33%	11%	46%	7%		
Usual Activities	No problems	58%	77%	52%	71%	9.265	**0.026**	67%	88%	49%	79%	26.866	** < 0.001**
	Problems	42%	23%	48%	29%			33%	12%	51%	21%		
Pain or Discomfort	No problems	63%	61%	43%	54%	5.108	0.164	67%	77%	51%	64%	10.553	**0.014**
	Problems	37%	39%	57%	46%			33%	23%	49%	36%		
Worried, Sad or Unhappy	No problems	74%	84%	70%	89%	6.507	0.089	69%	77%	65%	71%	2.645	0.450
	Problems	26%	16%	30%	11%			31%	23%	35%	29%		
VAS	Median (IQR)	100 (100;100)	93 (58;100)	100 (60;100)	100 (90;100)	8.032	**0.045**	88 (55;100)	99 (85;100)	100 (60;100)	100 (88;100)	6.549	0.088

Functional Disability (FD), General Population (GP), Orthopaedic condition (Ortho), Chronic Respiratory Condition (Resp). Bold scores indicate significance with a *p* value < 0.05

Although not contributing to significance the 5–7-year group, children with a functional disability or orthopaedic condition reported more problems than the general population and chronic respiratory group. Similarly the children in the younger group with an orthopaedic condition reported more *Pain or Discomfort* than the other groups.

The VAS scores were significantly different between the health groups for the 5–7-year group but not the 8–10-year group (Table 2). Notably the median VAS score was lowest for the general population group in the 5–7-year group, indicating worse general health. The VAS score in the 5–7-year-olds was significantly lower in the general population when compared to those with a chronic respiratory illness (H = 24.759 *p* = 0.016) and those with functional disability (H = 28.343, *p* = 0.023).

In the 8–10-year group, the VAS scores were significantly lower for children with a functional disability than the general population (H = 24.440, *p* = 0.039) and those with chronic respiratory illness (H = 33.577, *p* = 0.019).

### Convergent validity

The EQ-5D-Y-3L IA showed low to moderate convergent validity with individual items that were hypothesised to show an association and the dimension total scores on the WeeFIM, FPS-R and MFQ (Table 3). The dimension of *Looking After Myself* had significantly higher correlations with WeeFIM items of dressing and the self-care total in the 8–10-year-olds. Similarly, the dimension of *Worried, Sad or Unhappy* showed low significant correlations with MFQ items of unhappy, enjoyment and crying whereas younger children’s correlations was not significant. WeeFIM social interaction was not significantly associated with *Usual Activities* for either age-group.

**Table 3:** Convergent validity of the EQ-5D-Y-3L and corresponding items on the WeeFIM, Faces Pain Scale-Revised and Moods and Feelings Questionnaire

	5–7-years	8–10-years	5–7 vs 8–10-years	
	(n = 177)	(n = 211)	z-score	*p*-value
WeeFIM mobility	EQ-5D-Y-3L mobility			
Locomotion (walk/wheelchair for ≥ 45 m OR crawl ≥ 15 m)	− 0.51**	− 0.43**	− 1.03	0.151
Stairs climbing (ascend/descend 12–14 stairs)	− 0.43**	− 0.47**	0.49	0.312
Mobility total	− 0.38**	− 0.47**	1.07	0.142

WeeFIM self-care	EQ-5D-Y-3L Looking After Myself			
Grooming	− 0.27**	− 0.38**	1.20	0.115
Bathing (washing body excluding back)	− 0.61**	− 0.69**	1.35	0.089
Dressing upper body	− 0.42**	− 0.59**	2.24	**0.013**
Dressing lower body	− 0.40**	− 0.61**	2.78	**0.003**
Self-care total	− 0.54**	− 0.67**	2.01	**0.022**

WeeFIM mobility	EQ-5D-Y-3L Usual Activities			
Mobility total	− 0.28**	− 0.33**	0.54	0.295
Motor total^§^	− 0.36**	− 0.39**	0.34	0.369
*WeeFIM cognition*				
Social interaction (interaction with other children)	− 0.01	0.05	− 0.58	0.281

	EQ-5D-Y-3L Pain or Discomfort			
Faces Pain Scale-Revised	0.48**	0.38**	1.20	0.115

Moods and Feelings Questionnaire	EQ-5D-Y-3L Worried, Sad or Unhappy			
Unhappy	0.12	0.18**	− 0.60	0.274
Enjoyment	0.09	0.18**	− 0.89	0.187
Crying	0.08	0.21**	− 1.29	0.099
Lonely	0.21**	0.22**	− 0.10	0.460
Total	0.19*	0.34**	− 1.57	0.058

*Spearman’s correlation *p* < 0.05. **Spearman’s correlation *p* < 0.001, A significant z-score is bolded. A higher Moods and Feelings score, Faces Pain Scale-Revised score and EQ-5D-Y-3L score both indicate greater problems. A higher WeeFIM score indicates greater independence

### Test–retest reliability

In the younger group, *Mobility*, *Looking After Myself* and *Pain or Discomfort* showed significant, moderate test–retest reliability while *Usual Activities* and *Worried, Sad or Unhappy* showed significant, fair reliability (Table 4). In the older group, a significant, moderate reliability was found in *Mobility* with all other dimensions showing fair reliability. VAS scores across both age-groups were significant and reliable with an ICC > 0.70 while showing significant, fair agreements on Fleiss Kappa (k = 0.01–0.20). VAS scores showed a significant, weak agreement in 5–7-year-olds (Kendall’s W = 0.105) and no agreement in 8–10-year-olds (Kendall’s W = 0.105).

**Table 4:** Test–retest reliability of the EQ-5D-Y-3L across age-groups

		5–7-years	8–10-years
		(n = 177)	(n = 211)
Mobility	k	0.46**	0.50**
Looking After Myself	k	0.47**	0.38**
Usual Activities	k	0.27**	0.34**
Pain or Discomfort	k	0.42*	0.29*
Worried, Sad or Unhappy	k	0.27*	0.30*
VAS score	ICC	0.77**	0.70**
	Kendall’s W	0.105*	0.053*
	k	0.152*	0.138*

Dimension reliability k: Cohen's weighted Kappa, ICC: Intra-class correlation coefficient. VAS reliability k: Fleiss Kappa. **p > 0.00; *p < 0.05

### Cognitive debriefing

When asking the children the reasoning behind their responses, all responses for *Mobility* were logical and related to the physical activity of walking across all ages of children. For the dimension of *Looking After Myself* many who reported problems did so as they needed assistance, which was unrelated to their medical condition, this was significantly higher for the 5–7-year-olds (n = 70, 40%) than the 8–10-year-olds (n = 17, 8%) (*x*^2^ = 53.08, *p* < 0.001). This was most often attributed to needing assistance to dress, most notably with advanced dressing tasks such as buttons and laces with many reporting that they were currently still learning how to perform these tasks. For the dimension of *Usual Activities,* there were similar low reports of problems across the two age-groups that were unrelated to their medical condition (*x*^2^ = 0.53, *p* = 0.467). The reasons given would impact on their *Usual Activities* and included, bullying, fighting with siblings, and COVID-related restrictions. All reasons for experiencing *Pain or Discomfort* and *Worried, Sad or Unhappy* were related to the dimension with only one 8–10-year-old reporting *Pain or Discomfort* for emotional pain.

Significantly more 5–7-year-olds reported difficulty in understanding (n = 17, 10%) than the 8–10-year-olds (n = 8, 4%) (*x*^2^ = *4.47*, *p = 0.035*). Of the 5–7-year-olds reporting difficulty, one reported that he/she did not understand any of the questions asked. Reasons for difficulties experienced per age-group are shown in Table 5, most of which were associated with difficulty with certain words or comprehension of items.

**Table 5:** Reasons for difficulties reported with completion of the EQ-5D-Y-3L across age-groups and dimensions

Dimension/s	Reason	5–7-years (n = 17)		8–10-years (n = 8)	
		n	%	n	%
Mobility	I didn’t understand "about"	1	6	1	13
	I didn’t understand the question	3	18	0	0
	I had to think a lot	1	6	0	0
Looking After Myself	The question was difficult	1	6	0	0
	I didn’t understand the question	0	0	1	13
Usual Activities	There was a lot to think about	5	29	0	0
	I don’t enjoy those activities so I didn’t know what to say	0	0	1	13
	I don’t understand the question	1	6	0	0
Pain/Discomfort	I didn’t understand the words	1	6	2	25
	I didn’t understand "discomfort”	2	12	1	13
Worried, Sad or Unhappy	I wasn’t sure/can't explain how I was feeling	1	6	1	13
	I didn’t understand the words	1	6	0	0
	I didn’t know how to answer	0	0	1	13
	I had to think about it	1	6	0	0

Some children reported more than one difficulty

## Discussion

The EQ-5D-Y-3L IA showed similar convergent validity and test–retest reliability as children aged 8–10-years with similar health conditions and socioeconomic background. Many of the differences noted between the age-groups can be attributed to the developmental age of the child rather than a poor understanding of the concept or an inability to rate their health.

The time taken to complete the IA questionnaire was significantly longer for the younger children however, both questionnaires could be completed in under 2.5 min. This is not much longer than the 1 min completion time reported for self-complete in children aged 8–12-years [37] and is still feasible for administration in a clinical setting. The feasibility of the measure in the younger children was further shown with a similar ceiling at dimension level to older children, indicating similar ability to self-report on their health status. In accordance with other studies and as hypothesised, a higher ceiling effect was seen in the general population compared to other condition groups [18, 19, 23, 46–48]. The younger children did however report significantly fewer unique health states. This may result in a concentration of select health profiles and may negatively impact the ability to detect a change in the distribution of profile data over time and to compare profiles between children with different health conditions [49].

At a dimension level, there were no significant differences in reporting of problems in *Mobility* across age-groups. With similar findings to previous studies, South African children seem to perceive environmental barriers such as safety in their community to impact their mobility despite being physically able to mobilise [27] Cultural adaptation may be warranted to reflect that these problems are related to health rather than environmental or social circumstances. *Looking After Myself* had a higher report of problems in the younger children which was similarly found on the EQ-5D-Y-3L proxy in children 4–7-years-old [13]. This is further highlighted by the significant difference between age-groups for convergent validity with WeeFIM items of dressing. These problems were related to normal development with them reporting needing assistance with more advanced dressing tasks such as fastening buttons and tying shoelaces. Adaptation of the wording of this dimension may make it more appropriate for younger children [13]. Suggestions for adaptation should refer either to age appropriate dressing tasks and/or that the difficulties refer to a consequence of their health.

Considering the dimension of having *Pain or Discomfort,* there was a significantly higher report of problems by the 5–7-year-olds. However, this seemed to have been accurate with a moderate and significant correlation with the FPS-R and no difference with correlations between the age-groups report of *Pain or Discomfort.* The convergent validity with the WeeFIM and FPS-R was comparable across both age-groups to previous results reported by Scott et al. [27] for South African children aged 8–12-years. Thus, reporting of *Pain or Discomfort* seems to be easily reported across all age-groups and not affected by developmental abilities or level of schooling.

In keeping with previous studies, older children reported more problems on the of dimension of feeling *Worried, Sad or Unhappy* compared to younger children [28, 46]. These feelings were similar to previous research on the EQ-5D-Y-3L self-report and were largely associated with the child’s presenting medical condition or missing their family due to a hospital admission subsequent to their medical condition/injury [28, 46]. The convergent validity of the MFQ and *Worried, Sad or Unhappy* generally showed low and significant correlations across both age-groups which may be attributed to the difference in recall periods, with the MFQ using a two-week time frame and the EQ-5D-Y-3L IA refers to today. Despite young children understanding the concept of time, their ability to recall physical and psychosocial functioning lessens over time, therefore the recall period becomes incredibly important in younger children [50]. Furthermore, this could be attributed to the great variation in emotions experienced in a two-week period. Future research may consider comparing results to instruments with a similar time frame as the EQ-5D-Y, e.g. the CHU-9D, to reduce this effect [23]. The sample size for known-group validity in this study was small and the results should thus be interpreted with caution [38]. The dimension results for the 8–10-year group are in keeping with previous studies which reported significant differences between children with an acute health condition and those from the general population or with a chronic health condition [13, 27] across all dimensions except for *Worried, Sad or Unhappy* [19, 46, 51]*.* The dimension scores were not able to differentiate between health conditions in the 5–7-year group for the dimensions of *Mobility* and *Pain or Discomfort*. The trend was however similar to that of the older group and the insignificant result could be attributed to the relatively small sample of children or that younger children have more difficulty interpreting the dimensions and attributing them to their health. Of note, the general health score, as measured on the VAS, was lowest for children from the general population. It is unclear whether this younger age-group did not understand the VAS task and relation to general health or whether younger children, by nature, have greater dependence on their caregivers and thus poor health (as included in this study) has less impact. Known-group validity warrants further research with sufficiently sized samples in each known condition group [52] and with further investigation on the understanding of the VAS.

Children aged 5–7-years showed no systematic differences in test–retest reliability with similar reliability reported by Canaway and Frew [23] when using the EQ-5D-Y-3L self-complete version in children 6–7-years and in South African children aged 8–12-years [27]. Therefore proving that younger children were able to consistently understand and accurately interpret the EQ-5D-Y-3L to reflect on their health state on two different occasions. When considering individual dimension reliability this was consistent with previous reports for *Mobility, Looking After Myself* and *Pain or Discomfort* [18, 19, 23, 27]. *Looking After Myself* showed higher agreement for test–retest on self-complete in older children [18, 19, 27] while a lower agreement was found in 6–7-year-olds [23]. This is in keeping with the difficulty in completing the *Looking After Myself* dimension due to developmental age. Reasons for level of reporting was not taken at retesting although the health condition was postulated to remain stable across both age-groups.

There was little difficulty reported with understanding the EQ-5D-Y-3L IA across both age-groups, although there was more difficulty reported in the 5–7-year group, there was only one child aged 7-years who did not understand any of the questions. The most frequent reason for the difficulty in the 5–7-year group was that it required a lot of thinking. This was most notable for the dimension of *Usual Activities* which has more examples to remember through recall. Other reasons for difficulty included the unfamiliarity with some of the words used in the descriptive system (e.g. about and discomfort), this however did not impact the children’s understanding of what was being asked.

The general population group was recruited from the same geographical catchment area as the children from the tertiary paediatric hospital however, results cannot be generalised to the greater Western Cape region as no data on race, home language, and socioeconomic status was collected. This therefore limits the use and application of results across the greater Western Cape region.

At the time of data collection, only an English version of the EQ-5D-Y-3L IA was available, thus only English-speaking children were recruited. Considering South Africa has 11 official languages, many children who did not consider English as their home language were excluded and could not participate. Selection bias, as a result of only having an English version available, emphasises the need and recommendation for translations into other South African languages to ensure inclusion for all.

## Conclusion

The EQ-5D-Y-3L IA showed acceptable convergent validity and test–retest reliability for measuring health in a sample of English South African children aged 5–7-years. The performance of the measure was similar to children aged 8–10-years although there was more report of problems with the dimension of *Looking After Myself* due to younger children requiring help with advanced dressing tasks such as buttons and shoelaces therefore attributing these problems to developmental age rather than poor understanding of the dimension. There was further some reported difficulty with thinking about the dimensions in the younger age-group, most notably for *Usual Activities* in which the large number of examples may be too complex for younger children to report on. Adaptations to the dimensions of *Looking After Myself* and doing *Usual Activities* could improve the suitability of the EQ-5D-Y-3L to interviewer-administration in younger children.

Future research is encouraged to include a larger sample per group to establish known group validity  and further explore the understanding of the VAS in children aged 5–7-years. Further research into the responsiveness of the EQ-5D-Y-3L IA is recommended to determine its ability to detect change in paediatric health status over time. Psychometric testing and cognitive debriefing of the EQ-5D-Y-3L in children aged 5–7-years is recommended across different cultures, languages and literacy levels.

## Data Availability

The datasets used and/or analysed during the current study are available from the corresponding author on reasonable request.

## Abbreviations

FPS-R: Faces Pain Scale-Revised
HRQoL: Health-related Quality of Life
IA: Interviewer-Administered
ICC: Intraclass Correlation
IQR: Interquartile Range
MFQ: Moods and Feelings Questionnaire
PROM: Patient-reported Outcome Measure
VAS: Visual Analogue Scale
